# The Impact of a 10-Month Synbiotic Intake on eGFR, Uremic Toxins, Oxidative Stress, and Inflammatory Markers in Non-Dialysis Chronic Kidney Disease Patients: A Prospective, Non-Randomized, Placebo-Controlled Study

**DOI:** 10.3390/medicina61071199

**Published:** 2025-06-30

**Authors:** Teodor Kuskunov, Eduard Tilkiyan, Irina Zdravkova, Siyana Valova, Krasimir Boyanov, Anelia Bivolarska

**Affiliations:** 1 Department of Propaedeutics of Internal Diseases, Medical Faculty, Medical University of Plovdiv, 4000 Plovdiv, Bulgaria; irinazdravkova@abv.bg; 2 Hemodialysis Unit, University Hospital “Kaspela”, 4000 Plovdiv, Bulgaria; 3 Second Department of Internal Diseases, Section “Nephrology”, Medical Faculty, Medical University of Plovdiv, 4000 Plovdiv, Bulgaria; eet64@yahoo.com (E.T.); siyana.valova@mu-plovdiv.bg (S.V.); 4 Nephrology Clinic, University Hospital “Kaspela”, 4000 Plovdiv, Bulgaria; 5 Department of Medical Biochemistry, Faculty of Pharmacy, Medical University of Plovdiv, 4000 Plovdiv, Bulgaria; krasimir.boyanov@mu-plovdiv.bg (K.B.); anelia.bivolarska@mu-plovdiv.bg (A.B.)

**Keywords:** chronic kidney disease, indoxyl sulfate, p-cresyl sulfate, interleukin-6, malondialdehyde, synbiotic

## Abstract

*Background and Objectives*: The worldwide prevalence of chronic kidney disease (CKD) continues to increase, representing a major concern for public health systems. CKD is associated with gut microbiota dysbiosis, which may exacerbate disease progression by increasing the levels of uremic toxins, systemic inflammation, and oxidative stress. Modulation of the gut microbiota through biotic supplementation has been proposed as a potential therapeutic strategy to slow CKD progression and mitigate its complications. This study aimed to evaluate the effect of 10-month synbiotic supplementation on estimated glomerular filtration rate (eGFR), circulating concentrations of indoxyl sulfate (IS), p-cresyl sulfate (p-CS), interleukin-6 (IL-6), and malondialdehyde (MDA) in patients with stage IV–V CKD not receiving dialysis, in comparison to placebo. *Materials and Methods*: Fifty non-dialysis CKD IV–V patients were assigned (n = 25 each) via matched, non-randomized allocation (age, sex, and primary disease) to synbiotic or placebo. This single-blind, placebo-controlled trial blinded participants and laboratory personnel. The synbiotic group received daily capsules containing *Lactobacillus acidophilus* La-14 (2 × 10^11^ CFU/g) + fructooligosaccharides; controls received identical placebo. Adherence was monitored monthly (pill counts, diaries), with < 80% over two visits resulting in withdrawal. The eGFR, IS, p-CS, IL-6, and MDA were measured at baseline and month 10. *Results*: Forty-two patients (21/arm) completed the study; eight withdrew (4 per arm). At 10 months, the change in eGFR was −1.2 ± 2.5 mL/min/1.73 m^2^ (synbiotic) vs. −3.5 ± 3.0 mL/min/1.73 m^2^ (placebo); between-group difference in change was 2.3 mL/min/1.73 m^2^ (95% CI: 0.5–4.1; *p* = 0.014; adjusted *p* = 0.07). IS decreased by −15.4 ± 8.2 ng/L vs. −3.1 ± 6.5 ng/L; between-group difference in change was −12.3 ng/L (95% CI: −17.8 to −6.8; *p* < 0.001; adjusted *p* = 0.005). No significant differences were observed for p-CS, IL-6, or MDA after correction. *Conclusions*: Synbiotic supplementation over a 10-month period resulted in a trend toward decreased serum IS levels in patients with advanced CKD, suggesting potential benefits of microbiota-targeted therapies. However, no significant effects were observed on renal function, inflammatory, or oxidative stress markers. Further large-scale studies are warranted to confirm these findings and explore the long-term impact of synbiotics in CKD management.

## 1. Introduction

The human gastrointestinal tract hosts over 100 trillion microbial cells, collectively forming the gut microbiota. This intricate and ever-evolving microbial ecosystem is essential for preserving health and is involved in the development of numerous diseases. It is believed that each individual harbors a unique gut microbiota, comprising approximately 500 to 1000 bacterial species [1]. Microbial colonization of the gastrointestinal tract starts at birth and gradually matures, typically reaching a stable configuration between ages three and five [2]. In healthy individuals, approximately 90% of gut microbiota are represented by the phyla *Bacteroidetes*, *Firmicutes*, and *Actinobacteria*.

Within the framework of chronic kidney disease (CKD), the composition and functionality of the gut microbiota are altered, shifting from a symbiotic to a dysbiotic state [3,4]. Uremic patients frequently exhibit excessive colonization of the duodenum and jejunum by aerobic bacteria, predominantly *Enterobacteriaceae* and *Enterococci*, alongside reduced populations of beneficial species such as *Bifidobacterium* and *Lactobacillus* [5]. Additionally, an increased abundance of *Clostridium perfringens* has been reported in CKD patients. Ren et al. [6] observed elevated levels of *Thalassospira*, *Akkermansia*, and *Blautia* in fecal samples from CKD patients, while *Mollicutes RF9_norank* was significantly reduced. Notably, *Akkermansia* abundance correlated positively with serum creatinine and urea, suggesting its potential as a diagnostic or therapeutic marker in CKD.

Wang et al. [7] identified *Eggerthella lenta* and *Fusobacterium nucleatum*—microorganisms involved in indole and phenol production—as predominant species in end-stage renal disease (ESRD). The development of intestinal dysbiosis in CKD is thought to result from increased urea secretion into the gastrointestinal tract, which assumes a predominant role in excretion as the kidney function deteriorates [8]. Urea migrates from the circulation into the gut lumen, where microbial urease catalyzes its conversion into ammonia and subsequently ammonium hydroxide (NH_4_OH) [9]. This reaction raises intestinal pH, promotes overgrowth of urease-producing bacteria, impairs the proliferation of commensal microbes, and disrupts mucosal integrity, thereby exacerbating dysbiosis.

Elevated levels of ammonia and NH_4_OH damage the intestinal epithelium and compromise barrier function, resulting in increased gut permeability [10]. Such alterations impair carbohydrate fermentation and diminish the production of health-promoting short-chain fatty acids (SCFAs), including acetate, propionate, and butyrate. SCFAs are essential for maintaining intestinal integrity, modulating immune responses, supporting energy metabolism, and promoting cardiovascular health [11,12].

Disruption of the intestinal barrier permits the translocation of bacteria and endotoxins into systemic circulation, activating innate immune responses and provoking local and systemic inflammation [13]. In CKD, microbial dysbiosis fosters the overproduction of gut-derived uremic toxins, particularly indoxyl sulfate (IS; 213 Da) and p-cresyl sulfate (p-CS; 188 Da) [14,15]. These protein-bound solutes are normally excreted via tubular secretion; however, in CKD, impaired clearance results in their systemic accumulation [16].

IS and p-CS originate from the microbial metabolism of aromatic amino acids: Tryptophan is converted into indole by bacteria such as *Escherichia coli*, while tyrosine and phenylalanine are metabolized into p-cresol in the distal colon. Upon absorption, these precursors are transported to the liver, where they are sulfated into IS and p-CS and then excreted in the urine under normal physiological conditions [17].

The accumulation of these uremic toxins has been implicated in CKD progression and its complications. Indoxyl sulfate and p-cresyl sulfate promote oxidative stress by enhancing the production of reactive oxygen species (ROS) within endothelial cells [18,19]. ROS, in turn, interact with polyunsaturated fatty acids, leading to lipid peroxidation and the production of malondialdehyde (MDA), a biomarker of oxidative stress in CKD [20].

The intestinal microbiota also influence immune function, modulating cytokine production and immune cell activation. This process sustains a chronic, low-grade inflammatory state, marked by increased levels of IL-1β, IL-6, and various other pro-inflammatory mediators [21]. IL-1β has been shown to participate in tubulointerstitial fibrosis [22], while the soluble urokinase plasminogen activator receptor (suPAR), produced by activated immune cells, is associated with systemic inflammation and accelerated renal decline [23,24].

Moreover, indoxyl sulfate facilitates the epithelial-to-mesenchymal transition in proximal tubule cells, thereby advancing renal fibrotic processes [25]. The p-CS has been shown to induce apoptosis in tubular cells via upregulation of the TWEAK receptor Fn14 [26] and to suppress immune responses by inhibiting IFN-γ and IL-12 production [27]. The pivotal role of inflammation in CKD progression is supported by the CRIC study [28], which demonstrated that high circulating concentrations of IL-1β, IL-6, and TNF-α are associated with an accelerated deterioration in renal function.

Beyond renal damage, IS and p-CS are implicated in cardiovascular events, cognitive impairment, mineral and bone disorders, and neuroendocrine dysfunction in CKD patients. Modulating the gut microbiota through dietary interventions, probiotics, prebiotics, synbiotics, or fecal microbiota transplantation have emerged as potential therapeutic strategies. However, concerns regarding adverse effects, drug interactions, variability in microbiota composition, and long-term safety necessitate further investigation.

Consequently, comprehensive multicenter trials are crucial for advancing our insight into the gut–kidney axis and for designing effective microbiota-based treatments that offer a more comprehensive and effective approach to managing chronic kidney disease.

## 2. Materials and Methods

A total of 50 individuals (24 females and 26 males) diagnosed with stage IV–V CKD and not undergoing hemodialysis were enrolled between February and April 2023. The average age of participants was 58.1 ± 12.4 years. The study received approval from the Scientific Ethics Committee at the Medical University of Plovdiv (Protocol No. 1/19.01.2023), and written informed consent was obtained from all participants. All laboratory procedures were conducted at the Department of Medical Biochemistry, Medical University of Plovdiv. Participants were instructed to maintain their usual dietary habits, physical activity, and medication regimens throughout the study duration.

### 2.1. Study Design and Sample Size

Study Design: This was a prospective, single-blind, non-randomized, placebo-controlled study. Participants were blinded to group assignment: all capsules (synbiotic or placebo) were identical in appearance and packaging. Due to the matched, non-randomized allocation based on age, sex, and CKD etiology, investigators were not blinded and were responsible for dispensing capsules and monitoring follow-up. Laboratory personnel performing biomarker assays were blinded to group assignment.

Sample Size Rationale: The recruitment target of 50 participants (25 per arm) was based on feasibility and the number of eligible CKD stage IV–V patients available during the enrollment window. No formal a priori power calculation was performed. With 21 completers per group (n = 42), our study would have approximately 80% power to detect a large effect size (Cohen’s d ≥ 0.8) at α = 0.05 but is underpowered for smaller effects (d < 0.5). Therefore, null findings should be interpreted with caution.

### 2.2. Inclusion and Exclusion Criteria

Inclusion Criteria:

- Age ≥ 18 years;
- Diagnosed CKD stage IV–V not receiving dialysis;
- Ability to provide informed consent.

Exclusion Criteria:

- Inability to provide informed consent;
- Active inflammatory disease;
- Use of medications affecting gut microbiota (e.g., antibiotics, chemotherapy, biological agents) within the previous 3 months;
- Use of phosphate binders, statins, or proton pump inhibitors within 3 months.

### 2.3. Group Allocation

Participants were allocated to the synbiotic or placebo group using a matched allocation strategy based on age (±5 years), sex, and primary CKD etiology (e.g., diabetic nephropathy, hypertensive nephropathy, chronic glomerulonephritis, tubulointerstitial nephritis, and polycystic kidney disease). This matching aimed to ensure comparable baseline characteristics. Allocation was performed manually by the investigators at the time of enrollment.

### 2.4. Intervention and Compliance Monitoring

Intervention:

- Synbiotic group (n = 25): One gelatin capsule daily containing 75 mg *Lactobacillus acidophilus* La-14 (2 × 10^11^ CFU/g) plus 65 mg fructooligosaccharides, taken one hour after a meal for 10 months.
- Placebo group (n = 25): One identical gelatin capsule daily containing corn starch, taken one hour after a meal for 10 months.

Compliance Monitoring:

Participants received a 30-capsule supply each month. At each monthly visit, unused capsules were returned and counted by investigators (pill count). Participants also completed a daily intake diary, recording whether they took each capsule. Investigators reviewed diaries and pill counts to calculate adherence as:

Adherence (%) = (Capsules taken/Capsules dispensed) × 100.

Participants with adherence < 80% over any three consecutive months were counseled. If adherence remained < 80% at the subsequent visit, they were withdrawn for “poor compliance.” In total, three participants (two in the synbiotic group, one in placebo) were withdrawn due to poor compliance.

### 2.5. Blinding

This was a single-blind trial. Participants did not know whether they received synbiotic or placebo, as both capsules were identical in size, color, and taste. Investigators were aware of group assignments to manage dispensing and follow-up. Laboratory personnel performing assays for eGFR, uremic toxins, oxidative stress, and inflammatory markers were blinded to allocation.

### 2.6. Data Collection and Laboratory Analyses

At baseline and after 10 months, the following were measured:

- eGFR (calculated by the CKD-EPI formula);
- Serum indoxyl sulfate (IS);
- Serum p-cresyl sulfate (p-CS);
- Serum interleukin-6 (IL-6);
- Serum malondialdehyde (MDA).

Blood Sampling:

Venous blood (5 mL) was collected from fasting patients (≥8 h without food), allowed to clot at room temperature, then centrifuged at 1000× *g* for 20 min. Serum was aliquoted and stored at −80 °C until analysis.

ELISA Kits:

All ELISA assays (IS, p-CS, IL-6, and MDA) were performed using commercially available kits from ELK Biotechnology (Wuhan, China), following the manufacturer’s instructions.

### 2.7. Statistical Analysis

All statistical analyses were conducted using SPSS version 25.0 (SPSS Inc., Chicago, IL, USA). Continuous variables were tested for normality using the Shapiro–Wilk test. Data are presented as mean ± standard deviation (SD) or, if non-normal, median (interquartile range). For each outcome, change (Δ) was defined as (10-month value)—(baseline value). Between-group comparisons of Δ (synbiotic vs. placebo) were performed using independent samples *t*-tests if normally distributed or Mann–Whitney U tests if not. Within-group changes were assessed with paired *t*-tests or Wilcoxon signed-rank tests as appropriate. Because five primary/secondary outcomes were evaluated (eGFR, IS, p-CS, IL-6, and MDA), the Bonferroni correction was applied: significance threshold = 0.05/5 = 0.01. All tests were two-tailed, and *p* < 0.01 was considered statistically significant after correction.

## 3. Results

### 3.1. Baseline Demographic and Clinical Characteristics of the Two Subgroups Are Summarized in Table 1, Table 2 and Table 3: There Were No Significant Differences Between Synbiotic and Placebo Arms in Terms of Age, Gender Distribution, CKD Etiology, Comorbidities, or Baseline Biomarker Levels

A total of 21 patients in the synbiotic subgroup completed the study. Two patients initiated hemodialysis, and two others discontinued participation due to poor compliance. Similarly, 21 patients in the placebo subgroup completed the trial, with 3 beginning renal replacement therapy (RRT) and 1 withdrawing voluntarily. No adverse events were reported in either group during the follow-up period.

**Table 1:** Distribution of patients in the two subgroups according to age and gender.

Variable	Total (n = 50)	Synbiotic (n = 25)	Placebo (n = 25)	*p*-Value (Between Groups)
Age (years), mean ± SD	58.1 ± 12.4	59.0 ± 12.8	57.2 ± 12.0	0.62
Female, n (%)	25 (50%)	13 (52%)	12 (48%)	1.00
Male, n (%)	25 (50%)	12 (48%)	13 (52%)	1.00

SD—standard deviation.

**Table 2:** Distribution of patients based on CKD etiology and comorbidities in the two subgroups.

Variable	Total (n = 50)	Synbiotic (n = 25)	Placebo (n = 25)
CKD Etiology, n (%)			
• Chronic glomerulonephritis	16 (32%)	8 (32%)	8 (32%)
• Hypertensive nephropathy	13 (26%)	6 (24%)	7 (28%)
• Diabetic nephropathy	11 (22%)	6 (24%)	5 (20%)
• Chronic tubulointerstitial nephritis	6 (12%)	3 (12%)	3 (12%)
• ADPKD	2 (4%)	1 (4%)	1 (4%)
• Other	2 (4%)	1 (4%)	1 (4%)
Comorbidities, n (%)			
• AHT + IHD	18 (36%)	10 (40%)	8 (32%)
• DM + AHT	15 (30%)	7 (28%)	8 (32%)
• AHT + CVD	6 (12%)	3 (12%)	3 (12%)
• AHT only	8 (16%)	4 (16%)	4 (16%)
• None	3 (6%)	1 (4%)	2 (8%)

CTIN—Chronic tubulointerstitial nephritis, ADPKD—Autosomal dominant polycystic kidney disease, AHT—arterial hypertension, IHD—ischemic heart disease, DM—diabetes mellitus, and CVD—cerebrovascular disease.

**Table 3:** Baseline clinical characteristics.

Variable	Total (n = 50)	Synbiotic (n = 25)	Placebo (n = 25)	*p*-Value (Between Groups)
eGFR (mL/min/1.73 m^2^), mean ± SD	16.4 ± 5.8	16.5 ± 6.2	16.3 ± 5.3	0.96
IS (ng/L), mean ± SD	128.3 ± 30.2	135.9 ± 39.8	120.7 ± 20.6	0.07
p-CS (pg/mL), mean ± SD	15.3 ± 10.0	15.8 ± 11.2	14.8 ± 10.6	0.78
IL-6 (pg/mL), mean ± SD	17.7 ± 11.3	17.1 ± 13.6	18.3 ± 8.2	0.81
MDA (pg/mL), mean ± SD	1739.0 ± 706.9	1712.1 ± 721.3	1765.9 ± 691.5	0.86

### 3.2. Changes in eGFR and Uremic Toxins

In the synbiotic group, the mean eGFR decreased non-significantly from 16.5 ± 6.2 to 15.6 ± 6.7 mL/min/1.73 m^2^ (*p* = 0.12), whereas the placebo group experienced a significant decline from 16.3 ± 5.3 to 15.0 ± 5.8 mL/min/1.73 m^2^ (*p* < 0.001). The between-group difference in change in eGFR was 2.3 mL/min/1.73 m^2^ (95% CI: 0.5–4.1; *p* = 0.014; adjusted *p* = 0.07). Serum IS levels decreased significantly in the synbiotic group (−15.4 ± 8.2 µg/L; *p* = 0.019), but not in placebo (−3.1 ± 6.5 µg/L; *p* = 0.52). The between-group difference in change in IS was −12.3 µg/L (95% CI: −17.8 to −6.8; *p* < 0.001; adjusted *p* = 0.005) (Table 4).

Between-group comparisons: change in eGFR = 2.3 mL/min/1.73 m^2^ (95% CI: 0.5 to 4.1; *p* = 0.014; adjusted *p* = 0.07), change in IS = −12.4 ng/L (95% CI: −17.8 to −6.8; *p* < 0.001; adjusted *p* = 0.005). There were no statistically significant changes in p-cresyl sulfate, interleukin-6, or malondialdehyde levels from baseline to month 10 within or between the synbiotic and placebo groups. All within-group and between-group p-values exceeded 0.05, and no differences remained significant after Bonferroni correction (α = 0.01) (Table 5).

## 4. Discussion

Objectives and Novelty:

This study was designed to assess, for the first time in our center, the impact of 10 months of daily synbiotic supplementation on renal function (eGFR), circulating indoxyl sulfate (IS), p-cresyl sulfate (p-CS), interleukin-6 (IL-6), and malondialdehyde (MDA) in patients with stage IV–V chronic kidney disease (CKD) not yet on dialysis. To our knowledge, it represents the second published trial (after SYNERGY II [29]) examining long-term (≥10 months) synbiotic intake in advanced non-dialysis CKD. Our primary hypothesis was that synbiotic therapy would slow eGFR decline and lower serum uremic toxins (IS and p-CS), while modestly reducing systemic inflammation and oxidative stress.

Main Findings:

1. eGFR decline: Over 10 months, the synbiotic group experienced a non-significant decrease in eGFR (Δ = −1.2 ± 2.5 mL/min/1.73 m^2^; *p* = 0.12), whereas placebo patients had a statistically significant decline (Δ = −3.5 ± 3.0 mL/min/1.73 m^2^; *p* < 0.001). The between-group difference in ΔeGFR was 2.3 mL/min/1.73 m^2^ (95% CI: 0.5 to 4.1; *p* = 0.014) but did not remain significant after Bonferroni correction (adjusted *p* = 0.07).
2. Indoxyl sulfate (IS): Synbiotic supplementation led to a significant reduction in serum IS (Δ = −15.4 ± 8.2 ng/L; *p* = 0.019), while the placebo group saw a non-significant rise (Δ = −3.1 ± 6.5 ng/L; *p* = 0.52). The between-group difference (−12.3 ng/L; 95% CI: −17.8 to −6.8; *p* < 0.001) remained significant after Bonferroni correction (adjusted *p* = 0.005).
3. p-Cresyl sulfate (p-CS), IL-6, and MDA: Neither p-CS, IL-6, or MDA changed significantly in either group, and between-group comparisons all fell short of significance (adjusted *p* > 0.10).

Comparison with Prior Studies:

- eGFR: Our trend toward a slower eGFR decline in synbiotic-treated patients echoes the majority of meta-analyses showing no robust improvement in renal function from biotic supplements in CKD [30,31,32,33,34]. For example, Pisano et al. [30] (n = 345, stages II–V) and Firouzi et al. [31] (n = 376 stage II–V) both concluded that biotics did not meaningfully alter eGFR. McFarlane et al. [32] similarly found no eGFR benefit in three pooled RCTs. SYNERGY II, a 12-month, synbiotic RCT in CKD III–V, actually reported a significant eGFR drop in the intervention arm [29]. In our trial, although the unadjusted between-group *p* = 0.014 suggests a smaller decline with synbiotic, the effect size (≈2.3 mL/min/1.73 m^2^) was modest and lost statistical significance after correction, indicating limited clinical relevance.
- Indoxyl sulfate: The significant IS reduction in our synbiotic group aligns with shorter trials (4–12 weeks) showing lowered IS after prebiotic or synbiotic intake [33,35,36] mainly in hemodialysis patients, but it contrasts with others’ null toxin results [29,32,34]. The magnitude of IS lowering (≈12 ng/L difference) in our CKD IV–V cohort suggests that extended synbiotic dosing can reduce protein-bound toxins even in advanced CKD, provided sufficient duration and adherence.
- p-Cresyl sulfate: We observed no p-CS change, consistent with most non-dialysis trials and meta-analyses [29,32,34], indicating that p-CS is more refractory to modulation outside of dialysis settings [35,36].
- Inflammation (IL-6) and Oxidative Stress (MDA): Our non-significant reductions in IL-6 and MDA are in line with Liu et al. [37] and Yu Z et al. [38] who reported that only prebiotics (rather than synbiotics) achieved a consistent IL-6 drop. Zheng et al. [39] tended to MDA decreases only with longer (>12 weeks) interventions in select subgroups (with lower BMI). In dialysis patients, our prior single-arm 8-week study [35] found significant IL-6 and MDA decreases, suggesting that synbiotics may exert stronger anti-inflammatory/antioxidant effects in hemodialysis patients.

Interpretation and Mechanistic Insights:

The selective reduction in IS (but not p-CS) likely reflects the fact that IS derives primarily from bacterial tryptophan metabolism, while p-CS stems from tyrosine/phenylalanine. The synbiotic formula used (L. acidophilus La-14 plus fructooligosaccharides) may preferentially suppress indole-producing species (e.g., *Escherichia coli*, *Clostridium* spp.) or promote SCFA producers that compete metabolically. However, the absence of microbiome profiling in our protocol prevents us from confirming which taxa shifted. Likewise, modest or absent effects on IL-6 and MDA could reflect insufficient synbiotic potency to alter systemic inflammation or oxidative stress in the setting of advanced CKD, especially when residual kidney function remains low.

Strengths and Limitations:

- Strengths:

1. Duration and Stage: Ten months of observation in CKD IV–V is longer than most published synbiotic trials.
2. Single-Blind Design with Rigorous Adherence Tracking: Pill counts and diaries ensured that only adherent patients were analyzed.
3. Comprehensive Biomarkers: We measured both protein-bound uremic toxins and markers of inflammation/oxidative stress.

- Limitations:

1. Small Sample Size and Power: Only 42 completers (21 per arm) yielded ~80% power to detect large effect sizes (d ≥ 0.8). Consequently, null findings for p-CS, IL-6, and MDA may reflect a type II error rather than a true lack of effect.
2. Non-Randomized Allocation and Single-Blinding: Investigators knew group assignments, introducing potential performance bias. Laboratory staff were blinded, but unmeasured behavioral changes cannot be excluded.
3. No Microbiome Data: Without stool sequencing or metabolomics, our mechanistic interpretations remain speculative.
4. Absence of Pre-Registration: Primary and secondary outcomes were defined a priori in our internal protocol but were not registered, exposing us to possible reporting bias.
5. Withdrawals for Non-Compliance and RRT (n = 8): This further reduced sample size and may have introduced attrition bias.
6. Single-Center Study: Limits generalizability beyond our local CKD population.

Clinical Implications and Future Directions:

Our findings suggest that long-term synbiotic supplementation can meaningfully reduce serum IS in advanced CKD patients, even when eGFR preservation is marginal. Since IS is implicated in CKD progression and cardiovascular risk, lowering it may hold therapeutic value. However, given our modest sample and the absence of an eGFR benefit after correction, synbiotics should remain the adjunctive rather than the primary therapy. Future research should focus on:

1. Larger, randomized, double-blind RCTs in CKD III–V with block randomization to balance key covariates.
2. Microbiome and metabolomic profiling to link specific bacterial taxa or metabolic pathways to reductions in uremic toxins.
3. Longer follow-up (≥ 12 months) to determine if sustained IS lowering translates into slower progression (e.g., dialysis initiation, cardiovascular events).
4. Dose–response and formulation studies comparing different probiotic strains, prebiotic fibers, or synbiotic combinations to identify the most efficacious formula.
5. With the inclusion of earlier CKD stages (II–III), it is plausible that synbiotics are more effective when residual kidney function is higher.

## 5. Conclusions

In this prospective, single-blind, non-randomized, placebo-controlled study of synbiotic supplementation for 10 months in non-dialysis CKD stage IV–V patients, the results are as follows:

1. Primary Outcomes: Synbiotic intake significantly reduced serum indoxyl sulfate compared with placebo (between-group ΔIS = −12.3 ng/L; adjusted *p* = 0.005).
2. Renal Function: The synbiotic group showed a smaller eGFR decline than placebo (ΔeGFR = −1.2 vs. −3.5 mL/min/1.73 m^2^), but this difference lost statistical significance after the multiple comparison correction (adjusted *p* = 0.07) and was not clinically robust.
3. Secondary Outcomes: No significant effects were observed on p-cresyl sulfate, interleukin-6, or malondialdehyde.

Limitations: This study’s small sample size (n = 42 completers), non-randomized allocation, single-blinding, lack of microbiome profiling, and no formal trial registration limit the generalizability of our results. Eight participants (four per group) were withdrawn for poor compliance or starting RRT, further reducing power.

Implications: While synbiotic therapy appears safe and effectively lowers IS in advanced CKD, its impact on preserving renal function, reducing inflammation, or mitigating oxidative stress remains uncertain. Larger, randomized, double-blind, microbiome-integrated trials are essential to determine whether sustained reductions in uremic toxins translate into meaningful clinical benefits (e.g., delayed dialysis, fewer cardiovascular events).

## Figures and Tables

**Table 4:** Changes in eGFR and indoxyl sulfate (IS) from baseline to month 10 (n = 42).

Outcome	Group	Baseline (Mean ± SD)	Month 10 (Mean ± SD)	Change (Mean ± SD)	Within-Group *p*	Between-Group *p* (Adjusted)
eGFR (mL/min/1.73 m^2^)	Synbiotic	16.5 ± 6.2	15.6 ± 6.7	−1.2 ± 2.5	0.12	0.014 (0.07)
	Placebo	16.3 ± 5.3	15.04 ± 5.83	−3.5 ± 3.0	<0.001	
IS (ng/L)	Synbiotic	135.9 ± 39.8	113.12 ± 39.52	−15.4 ± 8.2	0.019	<0.001 (0.005)
	Placebo	120.7 ± 20.6	124.7 ± 19.7	−3.1 ± 6.5	0.52	

Between-group *p*-values are adjusted for five comparisons (α = 0.01, Bonferroni). Values in parentheses are adjusted *p*.

**Table 5:** Changes in p-cresyl sulfate, interleukin-6, and malondialdehyde from baseline to month 10 (n = 42).

Outcome	Group	Baseline (Mean ± SD)	Month 10 (Mean ± SD)	Change (Mean ± SD)	Within-Group *p*	Between-Group *p* (Adjusted)
p-CS (pg/mL)	Synbiotic	15.8 ± 11.2	15.1 ± 10.6	−0.6 ± 3.3	0.890	0.53 (1.00)
	Placebo	14.8 ± 10.6	15.1 ± 10.5	+0.3 ± 3.1	0.901	
IL-6 (pg/mL)	Synbiotic	17.1 ± 13.6	12.4 ± 13.6	−4.7 ± 7.2	0.370	0.46 (1.00)
	Placebo	18.3 ± 8.2	17.4 ± 11.7	−0.96 ± 4.8	0.821	
MDA (pg/mL)	Synbiotic	1712.1 ± 721.3	1654.1 ± 743.0	−58.0 ± 145.0	0.833	0.72 (1.00)
	Placebo	1765.9 ± 691.5	1768.2 ± 642.7	+2.3 ± 130.0	0.941	

All between-group *p*-values were > 0.05; after Bonferroni correction (α = 0.01), none reached significance.

## Data Availability

The data supporting the findings of this study are available upon reasonable request from the corresponding author. Public sharing of the data is restricted by national legislation.

## Abbreviations

CFU	colony forming units
CKD	chronic kidney disease
CRP	C–reactive protein
Da	Dalton
eGFR	estimated glomerular filtration rate
ELISA	enzyme-linked immunosorbent assay
ESRD	end-stage renal disease
IFN-γ	interferon gamma
IL	interleukin 6
IL-12	interleukin 12
IL-1β	interleukin-1β
IS	indoxyl sulfate
MDA	malondialdehyde
p-CS	p-cresyl sulfate
ROS	reactive oxygen species
RRT	renal replacement therapy
SCFAs	short-chain fatty acids
SD	standard deviation
SEM	standard error of mean
SuPAR	soluble urokinase-type plasminogen activator receptor
TNF-α	tumor necrosis factor–alpha
TWEAK Fn14	tumor necrosis factor-like weak inducer of apoptosis, fibroblast growth factor-inducible 14

## References

[1] Gevers D., Knight R., Petrosino J.F., Huang K., McGuire A.L., Birren B.W., Nelson K.E., White O., Methé B.A., Huttenhower C. (2012). The Human Microbiome Project: A community resource for the healthy human microbiome. PLoS Biol..

[2] Rodríguez J.M., Murphy K., Stanton C., Ross R.P., Kober O.I., Juge N., Avershina E., Rudi K., Narbad A., Jenmalm M.C. (2015). The composition of the gut microbiota throughout life, with an emphasis on early life. Microb. Ecol. Health Dis..

[3] Chen T.H., Cheng C.Y., Huang C.K., Ho Y.H., Lin J.C. (2023). Exploring the Relevance between Gut Microbiota-Metabolites Profile and Chronic Kidney Disease with Distinct Pathogenic Factor. Microbiol. Spectr..

[4] Vaziri N.D., Wong J., Pahl M., Piceno Y.M., Yuan J., DeSantis T.Z., Ni Z., Nguyen T.H., Andersen G.L. (2013). Chronic kidney disease alters intestinal microbial flora. Kidney Int..

[5] Voroneanu L., Burlacu A., Brinza C., Covic A., Balan G.G., Nistor I., Popa C., Hogas S., Covic A. (2023). Gut Microbiota in Chronic Kidney Disease: From Composition to Modulation towards Better Outcomes-A Systematic Review. J. Clin. Med..

[6] Ren Z., Fan Y., Li A., Shen Q., Wu J., Ren L., Lu H., Ding S., Ren H., Liu C. (2020). Alterations of the Human Gut Microbiome in Chronic Kidney Disease. Adv. Sci..

[7] Wang X., Yang S., Li S., Zhao L., Hao Y., Qin J., Zhang L., Zhang C., Bian W., Zuo L. (2020). Aberrant gut microbiota alters host metabolome and impacts renal failure in humans and rodents. Gut.

[8] Chi M., Ma K., Wang J., Ding Z., Li Y., Zhu S., Liang X., Zhang Q., Song L., Liu C. (2021). The Immunomodulatory Effect of the Gut Microbiota in Kidney Disease. J. Immunol. Res..

[9] Vaziri N.D., Goshtasbi N., Yuan J., Jellbauer S., Moradi H., Raffatellu M., Kalantar-Zadeh K. (2012). Uremic plasma impairs barrier function and depletes the tight junction protein constituents of intestinal epithelium. Am. J. Nephrol..

[10] Mishima E., Fukuda S., Mukawa C., Yuri A., Kanemitsu Y., Matsumoto Y., Akiyama Y., Fukuda N.N., Tsukamoto H., Asaji K. (2017). Evaluation of the impact of gut microbiota on uremic solute accumulation by a CE-TOFMS-based metabolomics approach. Kidney Int..

[11] Liu X.F., Shao J.H., Liao Y.T., Wang L.N., Jia Y., Dong P.J., Liu Z.Z., He D.D., Li C., Zhang X. (2023). Regulation of short-chain fatty acids in the immune system. Front. Immunol..

[12] Magliocca G., Mone P., Di Iorio B.R., Heidland A., Marzocco S. (2022). Short-Chain Fatty Acids in Chronic Kidney Disease: Focus on Inflammation and Oxidative Stress Regulation. Int. J. Mol. Sci..

[13] Stanford J., Charlton K., Stefoska-Needham A., Zheng H., Bird L., Borst A., Fuller A., Lambert K. (2021). Associations Among Plant-Based Diet Quality, Uremic Toxins, and Gut Microbiota Profile in Adults Undergoing Hemodialysis Therapy. J. Ren. Nutr..

[14] Rysz J., Franczyk B., Ławiński J., Olszewski R., Ciałkowska-Rysz A., Gluba-Brzózka A. (2021). The Impact of CKD on Uremic Toxins and Gut Microbiota. Toxins.

[15] Kim S.M., Song I.H. (2020). The clinical impact of gut microbiota in chronic kidney disease. Korean J. Intern. Med..

[16] Cigarran Guldris S., González Parra E., Cases Amenós A. (2017). Gut microbiota in chronic kidney disease. Nefrologia.

[17] Suchy-Dicey A.M., Laha T., Hoofnagle A., Newitt R., Sirich T.L., Meyer T.W., Thummel K.E., Yanez N.D., Himmelfarb J., Weiss N.S. (2016). Tubular Secretion in CKD. J. Am. Soc. Nephrol..

[18] Azevedo M.L., Bonan N.B., Dias G., Brehm F., Steiner T.M., Souza W.M., Stinghen A.E., Barreto F.C., Elifio-Esposito S., Pecoits-Filho R. (2016). p-Cresyl sulfate affects the oxidative burst, phagocytosis process, and antigen presentation of monocyte-derived macrophages. Toxicol. Lett..

[19] Yan Z., Shao T. (2024). Chronic Inflammation in Chronic Kidney Disease. Nephron.

[20] Demirci-Çekiç S., Özkan G., Avan A.N., Uzunboy S., Çapanoğlu E., Apak R. (2022). Biomarkers of Oxidative Stress and Antioxidant Defense. J. Pharm. Biomed. Anal..

[21] Wastyk H.C., Fragiadakis G.K., Perelman D., Dahan D., Merrill B.D., Yu F.B., Topf M., Gonzalez C.G., Van Treuren W., Han S. (2021). Gut-microbiota-targeted diets modulate human immune status. Cell.

[22] Lemos D.R., McMurdo M., Karaca G., Wilflingseder J., Leaf I.A., Gupta N., Miyoshi T., Susa K., Johnson B.G., Soliman K. (2018). Interleukin-1*β* Activates a MYC-Dependent Metabolic Switch in Kidney Stromal Cells Necessary for Progressive Tubulointerstitial Fibrosis. J. Am. Soc. Nephrol..

[23] Hahm E., Wei C., Fernandez I., Li J., Tardi N.J., Tracy M., Wadhwani S., Cao Y., Peev V., Zloza A. (2017). Bone marrow-derived immature myeloid cells are a main source of circulating suPAR contributing to proteinuric kidney disease. Nat. Med..

[24] Hayek S.S., Sever S., Ko Y.A., Trachtman H., Awad M., Wadhwani S., Altintas M.M., Wei C., Hotton A.L., French A.L. (2015). Soluble Urokinase Receptor and Chronic Kidney Disease. N. Engl. J. Med..

[25] Bolati D., Shimizu H., Higashiyama Y., Nishijima F., Niwa T. (2011). Indoxyl sulfate induces epithelial-to-mesenchymal transition in rat kidneys and human proximal tubular cells. Am. J. Nephrol..

[26] Poveda J., Sanchez-Niño M.D., Glorieux G., Sanz A.B., Egido J., Vanholder R., Ortiz A. (2014). p-cresyl sulphate has pro-inflammatory and cytotoxic actions on human proximal tubular epithelial cells. Nephrol. Dial. Transplant..

[27] Shiba T., Kawakami K., Sasaki T., Makino I., Kato I., Kobayashi T., Uchida K., Kaneko K. (2014). Effects of intestinal bacteria-derived p-cresyl sulfate on Th1-type immune response in vivo and in vitro. Toxicol. Appl. Pharmacol..

[28] Amdur R.L., Feldman H.I., Gupta J., Yang W., Kanetsky P., Shlipak M., Rahman M., Lash J.P., Townsend R.R., Ojo A. (2016). Inflammation and Progression of CKD: The CRIC Study. Clin. J. Am. Soc. Nephrol..

[29] McFarlane C., Krishnasamy R., Stanton T., Savill E., Snelson M., Mihala G., Kelly J.T., Morrison M., Johnson D.W., Campbell K.L. (2021). Synbiotics Easing Renal Failure by Improving Gut Microbiology II (SYNERGY II): A Feasibility Randomized Controlled Trial. Nutrients.

[30] Pisano A., D’Arrigo G., Coppolino G., Bolignano D. (2018). Biotic Supplements for Renal Patients: A Systematic Review and Meta-Analysis. Nutrients.

[31] Firouzi S., Haghighatdoost F. (2018). The effects of prebiotic, probiotic, and synbiotic supplementation on blood parameters of renal function: A systematic review and meta-analysis of clinical trials. Nutrition.

[32] McFarlane C., Ramos C.I., Johnson D.W., Campbell K.L. (2019). Prebiotic, Probiotic, and Synbiotic Supplementation in Chronic Kidney Disease: A Systematic Review and Meta-analysis. J. Ren. Nutr..

[33] Liu J., Zhong J., Yang H., Wang D., Zhang Y., Yang Y., Xing G., Kon V. (2022). Biotic Supplements in Patients With Chronic Kidney Disease: Meta-Analysis of Randomized Controlled Trials. J. Ren. Nutr..

[34] Liu C., Yang L., Wei W., Fu P. (2024). Efficacy of probiotics/synbiotics supplementation in patients with chronic kidney disease: A systematic review and meta-analysis of randomized controlled trials. Front. Nutr..

[35] Kuskunov T., Tilkiyan E., Doykov D., Boyanov K., Bivolarska A., Hristov B. (2023). The Effect of Synbiotic Supplementation on Uremic Toxins, Oxidative Stress, and Inflammation in Hemodialysis Patients-Results of an Uncontrolled Prospective Single-Arm Study. Medicina.

[36] Takkavatakarn K., Wuttiputinun T., Phannajit J., Praditpornsilpa K., Eiam-Ong S., Susantitaphong P. (2021). Protein-bound uremic toxin lowering strategies in chronic kidney disease: A systematic review and meta-analysis. J. Nephrol..

[37] Liu T., Wang X., Li R., Zhang Z.Y., Fang J., Zhang X. (2021). Effects of Probiotic Preparations on Inflammatory Cytokines in Chronic Kidney Disease Patients: A Systematic Review and Meta-Analysis. Curr. Pharm. Biotechnol..

[38] Yu Z., Zhao J., Qin Y., Wang Y., Zhang Y., Sun S. (2022). Probiotics, Prebiotics, and Synbiotics Improve Uremic, Inflammatory, and Gastrointestinal Symptoms in End-Stage Renal Disease With Dialysis: A Network Meta-Analysis of Randomized Controlled Trials. Front. Nutr..

[39] Zheng H.J., Guo J., Wang Q., Wang L., Wang Y., Zhang F., Huang W.J., Zhang W., Liu W.J., Wang Y. (2021). Probiotics, prebiotics, and synbiotics for the improvement of metabolic profiles in patients with chronic kidney disease: A systematic review and meta-analysis of randomized controlled trials. Crit. Rev. Food Sci. Nutr..

